# Cognitive Profiles of Adolescent Inpatients with Substance Use Disorder

**DOI:** 10.3390/children9050756

**Published:** 2022-05-21

**Authors:** Angelika Beate Christiane Becker, Luisa Marie Lüken, Lea Kelker, Martin Holtmann, Monika Daseking, Tanja Legenbauer

**Affiliations:** 1 Department of Educational Psychology, Helmut-Schmidt-University/University of the Federal Armed Forces, 22043 Hamburg, Germany; lea.kelker@hsu-hh.de (L.K.); m.daseking@hsu-hh.de (M.D.); 2 LWL University Hospital for Child and Adolescent Psychiatry, Psychotherapy and Psychosomatics, Ruhr University Bochum, 59071 Hamm, Germany; luisa.lueken@uni-muenster.de (L.M.L.); martin.holtmann@lwl.org (M.H.); tanja.legenbauer@lwl.org (T.L.); 3 Department of Psychology, University of Münster, 48149 Münster, Germany

**Keywords:** substance use disorder, cannabis, alcohol, cognitive profile, WISC-V, WAIS-IV, intelligence, Full Scale IQ, processing speed

## Abstract

The prevalence of substance abuse is high during adolescence, and several studies have linked the use of alcohol and cannabis in adolescence to different cognitive impairments. To investigate whether specific cognitive deficits can be observed in adolescents with substance use disorder (SUD), we compared the cognitive profiles of inpatient adolescents diagnosed with SUD to a control group matched for sex, age and educational status. The inpatient adolescents received diagnoses of cannabis use disorder, alcohol use disorder or both. We compared the WISC-V profiles of 22 inpatients (45.5% female, M_age_: 14.5; SD: 0.8) and the WAIS-IV profiles of 27 inpatients (44.4% female, M_age_: 17.1; SD: 0.9) to 49 matched control participants with no diagnosed SUD. At the time of testing, participants were hospitalized for treatment of their SUD and were abstinent for a period of at least 6 weeks. To gain greater power, we jointly analyzed the Verbal Comprehension Index, Working Memory Index, Processing Speed Index and Full Scale IQ as assessed by WISC-V and WAIS-IV. The clinical group performed significantly worse than the control group on all the above indices. When only the group of inpatients was observed, in a model with the factors sex, educational status, presence of a comorbid diagnosis of depression and the number of comorbid diagnoses, only the factor educational status was significantly associated with the Full Scale IQ, whereas the factors sex and a comorbid diagnosis of depression in this group were associated with the Processing Speed Index. The results show that adolescents diagnosed with SUD (cannabis and/or alcohol) display broad cognitive impairments after 6 weeks of abstinence. Future research is required to further explore the role of comorbid diagnoses.

## 1. Introduction

Adolescence is the developmental period where major psychological, physical and neurodevelopmental changes take place [1]. In this period, maturation changes in the brain contribute to age-specific behavior patterns such as risk taking and substance use [2,3,4]. The use of psychoactive substances such as alcohol, nicotine, caffeine or illegal drugs in adolescence is widespread. In fact, according to the 2019 European School Survey Project on Alcohol and other Drugs (ESPAD) [5], more than 79% of 15- to 16-year-olds had consumed alcohol at least once, and 17% of these students had consumed illegal drugs. In 2019, the 30-day prevalence rates for Europe in general were 47% for alcohol use and 16% for cannabis use. In Germany, a 2019 survey reported that the 30-day prevalence rate for alcohol consumed by adolescents was 37% [6]. Additionally, in Germany in 2019, 14,500 children and adolescents from age 10 to 18 had to be hospitalized for acute alcohol intoxication [7]. Notably, the rate of substance use increases from early to late adolescence [8].

This is concerning because the use of psychoactive substances can lead to substance use disorder (SUD) [9,10,11], and starting substance use at a younger age is associated with a higher risk of developing SUD later in life [12,13]. In Germany, this seems to be the case for 6% of adolescents who use alcohol and 2–3% of adolescents who use narcotics [14]. In the US, studies have shown that 13- to 18-year-olds who use psychotropic substances have a lifetime prevalence of 11.4% for abuse and addiction [15]. In Germany, cannabis use disorder seems to be the main reason for consulting a psychiatrist [16,17].

According to the ICD-10 (International Statistical Classification of Diseases and Related Health Problems, Tenth Revision), SUD is defined as a group of physical, behavioral and cognitive phenomena in which the use of a substance or a class of substances takes priority for the affected person over other behaviors that they previously valued more highly. While the ICD-10 differentiates between harmful substance use and substance dependence, the DSM-5 uses the term substance use disorder with a focus on a dimensional classification, whereby the severity of symptoms can be specified as mild, moderate or severe. The latest version of the ICD, the ICD-11, includes the term “hazardous substance use”, which describes a pattern of repeated substance use [18,19,20].

SUDs include a range of different behaviors with a variety of physical, psychological and social consequences [16]. Comorbid psychiatric diseases are very common and often precede the occurrence of a SUD [21]; studies have reported that SUDs are most often comorbid with disorders of social behavior, ADHD, depression, impulse control disorders and personality disorders [22,23]. Furthermore, substance abuse can trigger disorders such as psychosis [24,25,26]. In a review by Hall et al. (2020), daily use of highly potent cannabis by adolescents was associated with severe psychological problems such as psychosis, mania and suicidality [27]. In this review, daily cannabis use was also linked with poor cognitive functioning, which may affect educational attainment and occupational choice. In a different review by Townsend et al. (2007) [28], cannabis use was found to be associated with dropping out of school early (see also [29] for a review). Further, cognitive impairment in patients with SUD also seems to contribute to poorer treatment outcomes [30,31].

A co-occurrent SUD seems also to be a significant predictor for 1-year rehospitalization in adolescents with mental disorders (along with being admitted for a suicide attempt) [32]. Thus, there might be a need for more specialized psychosocial interventions or aftercare for patients with SUD. Therefore, it is important to measure the cognitive abilities of adolescents with SUD not only for research purposes but also in practice to find the best possible therapies and support for these patients’ future life paths.

### 1.1. The Influence of Substance Use and Abuse on Adolescents’ Cognitive Abilities

Studies examining the influence of substance use on adolescents’ cognitive abilities face several challenges, and results have been inconsistent. Such inconsistencies may be due to the extent to which factors such as preexisting cognitive deficits were considered [33,34,35], the degree to which studies controlled for further influencing factors, or the heterogeneity of the clinical sample. Another important factor is whether studies investigated neuropsychological changes longitudinally or on only one or two measurement time points. Several review studies have summed up the observed changes in cognitive domains following substance use and/or addiction in adolescents [27,36,37]. In the following, we summarize the findings from studies investigating how adolescents’ cognitive abilities are influenced by cannabis and/or alcohol.

Regarding cannabis use, a systematic review [38] showed that acute or chronic cannabis use led to deficits in verbal memory and to mixed results for working memory, decision-making and executive functions. Furthermore, a large meta-analysis [39] found that frequent or heavy cannabis use was associated with reduced cognitive functioning in adolescents and young adults. Different factors seem to influence how cannabis use affects adolescents’ general IQ [36]. Meier et al. (2018) [40] found in a longitudinal co-twin control study that short-term cannabis use in adolescence had no effect on general IQ or executive functions, and Mokrysz et al. (2016) [41] found no difference in IQ between adolescent cannabis users and a control group when smoking cigarettes was added as a control factor. However, several studies show that adolescent cannabis users demonstrate significant changes in their cognitive profiles. For example, Meier et al. (2012) [42] found that adolescent cannabis users had greater IQ declines and working memory impairments at the age of 38 than users that started their cannabis use after the age of 18. Further, Castellanos-Ryan et al. (2017) [43] associated early onset and frequent cannabis use with deficits in verbal IQ, and Becker et al. (2015) [44] reported that adolescent cannabis users had persistent verbal learning impairments. In another study based on a very large sample, Petker et al. (2019) [45] linked positive urinary tests for tetrahydrocannabinol with worse performance in episodic memory and processing speed and linked diagnosed cannabis use disorder with lower fluid intelligence. In a meta-analysis, Figueiredo et al. (2020) [46] showed correlations between chronic cannabis use and deficits in short- and long-term memory, attention, cognitive flexibility and impulsivity.

For alcohol, several studies have shown poorer working and verbal memory in adolescent alcohol drinkers [36,47,48]. Nguyen-Louie et al. (2016) [49] showed that binge-drinking adolescents have poorer performance in verbal learning and memory tasks than moderately drinking adolescents, and this effect might be mediated by dose. Alcohol abuse has also been linked to deficits in attention and changes in impulsivity [37]. Another study showed that in binge-drinking adolescents, identified deficits in working memory were still observable three years later [50]. In yet another report, Nguyen-Louie et al. (2017) [51] found that the age in which weekly alcohol consumption started was able to predict psychomotor speed, visual attention, cognitive inhibition and working memory, where earlier ages led to worse outcomes. While informative, these findings were mainly determined using large population samples, not clinical samples dealing with pathological alcohol use; studies with clinical samples of adolescent pathological alcohol use are sparse.

### 1.2. Cognitive Profiles in Adolescents with a SUD

Test batteries such as the Wechsler Adult Intelligence Scale—Fourth Edition (WAIS-IV; [52]) for participants aged 17 or older or the Wechsler Intelligence Scale for Children—Fifth Edition (WISC-V; [53]) for participants aged 16 or younger can be useful in clinical settings because the cognitive profiles of patients in different domains can be tested with a single test. They can also be used to detect distinctive features in a cognitive profile that could lead to problems (e.g., working memory deficits) or explain existing problems (e.g., learning problems). In comparison to the previous version, the Wechsler Intelligence Scale for Children—Fourth Edition (WISC-IV; [54]), the WISC-V [53] includes five primary indices (Verbal Comprehension Index [VCI], Visual Spatial Index [VSI], Fluid Reasoning Index [FRI], Working Memory Index [WMI], Processing Speed Index [PSI]) and the Full Scale IQ [FSIQ]). On the basis of confirmatory factorial analyses, the Perceptual Reasoning Index was split into the new VSI and the FRI, and new subtests were added to the test (Figure Weights, Visual Puzzles and Picture Span). Now, only seven subtests contribute to the FSIQ instead of the 10 included in the WISC-IV. The WAIS-IV and WISC-V share the VCI, WMI, PSI and the Full Scale IQ, and evidence suggests that the constructs measured by the WAIS-IV and the WISC-V can be interpreted similarly [55].

In adults, several studies have investigated the cognitive profiles of clinical groups with SUD using standardized test batteries, including the earlier Wechsler Adult Intelligence Scale-Third Edition (WAIS-III; [56]) [57,58] as well as the later WAIS-IV [52]. In one study, Braatveit et al. (2018) [59] used the WAIS-IV [52] to measure the IQs of adults with SUD and assessed the factors that contribute to their IQ variance. They found that learning and attention deficit/hyperactivity difficulties in childhood were directly related to adult IQ, while education had a mediating effect; they found no effect of substance abuse. In a different study, Sullivan et al. (2021) [60] examined the predictive validity of the WAIS-IV in predicting treatment completion in a clinical sample. They found significant differences between the clinical sample and the population norm in the Processing Speed Index and the Verbal Comprehension Index, but not for the Working Memory Index or the Perceptual Reasoning Index.

For investigating adolescents, studies have used the WISC-IV [54] for participants 16 or younger and the WAIS-IV [52] (for participants 17 or older) in patients with SUD to investigate, for example, the outcomes of a working memory training for adolescents with cannabis use disorder [61]. In one study, Latvala et al. (2009) [62] used a different scale—the Wechsler Adult Intelligence Scale-Revised (WAIS-R; [63])—to investigate verbal intellectual ability, psychomotor processing speed and verbal and visual working memory in young adults with SUD. They found that verbal intellectual ability was lower in the clinical sample, but only if the parental and young adults’ educational status were not considered. Yet, psychomotor processing was linked to the SUD independent of other factors. To our knowledge, no study so far has used the latest version, the WISC-V, to investigate cognitive functioning and profiles of adolescents with SUD. In addition, many studies with clinical samples have not considered comorbid diagnoses.

### 1.3. Aims

Given these research gaps, we investigated the cognitive profiles of a group of adolescent inpatients with chronic and extensive substance abuse. Because inpatients’ cognitive function may be influenced by comorbid diagnoses, we also took these into account. For the study, we matched a group of youth inpatients with substance use disorder to a control group of healthy adolescents specifically regarding age, sex and educational status and examined their cognitive profiles; our goal was to determine whether specific cognitive patterns could be seen in the clinical group. Based on previous research, we expected the clinical group to show deficits in the Full Scale IQ, the Verbal Comprehension Index, the Working Memory Index and the Processing Speed Index. We also investigated the potential impact of the number of comorbid diagnoses, a comorbid diagnosis of depression, sex or type of education.

## 2. Materials and Methods

### 2.1. Recruitment and Participant Flow

In the recruitment period between May 2018 and January 2020, 313 patients with SUD were treated in the hospital for child and adolescent psychiatry. Only patients who were tested with the German version of the WISC-V [53] or the German version of the WAIS-IV [52] during their hospitalization were included in the sample. Intelligence testing only took place in some of the patients in cases of clinical indication. The final sample comprised *N* = 50 patients with SUD.

### 2.2. Statistical Analyses

Statistical analyses were performed in SPSS [64]. To increase the sample size, group comparisons between the clinical group and the control group were also performed by pooling the indices shared between the WISC-V and WAIS-IV (namely, the FSIQ, VCI, WMI, PSI). A MANOVA was conducted with those four indices as dependent variables and the factor group (clinical group vs. control group) as the independent variable. Second, the results in the WISC-V and the WAIS-IV were tested separately with a MANOVA. For both the WISC-V and WAIS-IV analyses, the group (clinical group vs. control group) represented the independent variable. The five primary indices (VCI, VSI, FRI, WMI, PSI) and the FSIQ were selected as dependent variables for the WISC-V, and for the WAIS-IV the dependent variables were the four indices VCI, PRI, WMI and PSI and the FSIQ. The statistical alpha level was set below 0.05. Eta squared was calculated as the effect size for parametrical group comparisons, and the Cohen’s d effect size was calculated for comparisons of two groups. Effect sizes were classified according to Cohen (1988) [65] as small effects (*η*^2^ = 0.01; *d* = 0.20), moderate effects (*η*^2^ = 0.06; *d* = 0.50), and large effects (*η*^2^ = 0.14; *d* = 0.80). We also calculated multiple linear regressions to determine relations between the predictor variables sex (male vs. female), type of education (low vs. middle vs. high), depression (diagnosis vs. no diagnosis) and number of comorbid diagnoses on the outcome variables FSIQ, VCI, WMI and PSI. We also classified *R* and *R*^2^ according to Cohen (1988) [65] as small effects (*R* = 0.10; *R*^2^ = 0.02), moderate effects (*R* = 0.03; *R*^2^ = 0.13) and large effects (*R* = 0.05; *R*^2^ = 0.26).

To investigate the connection between the number of comorbid diagnoses and the index scores FSIQ, VCI, WMI and PSI, the clinical group was split by median split in a group that had two or fewer comorbid psychiatric diagnoses and a group that had three or more comorbid psychiatric diagnoses. The groups were compared by a MANOVA with the independent variable group (two or fewer diagnoses vs. three or more diagnoses) and the dependent variables FSIQ, VCI, WMI and PSI.

## 3. Results

### 3.1. Description of the Sample

Participants with an overall IQ < 70 were excluded from the sample (*n* = 1), and thus the final sample consisted of *N* = 49 participants. Participants had different substance use profiles, with most patients having a cannabis-related disorder (ICD10: F12, *n* = 43, 87.8%), followed by a mostly comorbid alcohol-related disorder (ICD10: F10, *n* = 20, 40.8%). Of the total sample, *n* = 4 (8.2%) also had a cocaine-related disorder (ICD10: F14), and *n* = 6 (12.2) had disorders resulting from the use of multiple drugs (ICD10: F19). At the time of testing, participants were at least 6 weeks abstinent. Participants aged 16 or younger were tested with the German version of the WISC-V [53], *n* = 22 (45.5% female; M_age_: 14.5; SD: 0.8), while those aged 17 or older were tested with the German version of the WAIS-IV [52], *n* = 27 (44.4% female; M_age_: 17.1, SD: 0.9). Testing with the WISC-V or WAIS-IV took place upon hospitalization of the inpatients, during the diagnostic process. A control sample was formed by selecting children and adolescents from the German WISC-V standardization sample and adults from the German WAIS-IV standardization sample that matched the clinical group according to age, sex and type of education (see Table 1).

A Kruskal–Wallis test yielded no significant differences between the groups regarding the type of education, X^2^(3) = 2.937, *p* = *n*.s. Further psychiatric diagnoses were available for the participants. Several patients had comorbid diagnoses (see Table 2), and *n* = 27 (55.1%) had a comorbid diagnosis of a depression.

### 3.2. Comparisons of Cognitive Profiles

To enlarge the statistical power, the FSIQ, VCI, WMI and PSI were observed together with no separation by testing. Descriptive statistics and results of the MANOVA are displayed in Table 3. See Appendix A and Table A1 for descriptive statistics of the primary index scores separately for the WISC-V and the WAIS-IV and their comparisons across groups. Here, significant group differences were seen for all indices, with the largest effects for the FSIQ and the VCI.

### 3.3. Predictors of the Cognitive Profile

In the next step, only the clinical group was considered further. Sex, type of education, depression and number of comorbid diagnoses were able to statistically significantly predict the FSIQ score, *F*(4, 31) = 3.44, *p* = 0.019, and the PSI score, *F*(4, 31) = 4.79, *p* = 0.004. The *R*^2^ for the model for FSIQ was 0.31 (adjusted *R*^2^ = 0.22), and the *R*^2^ for the PSI was 0.38 (adjusted *R*^2^ = 0.30), indicative of a high goodness-of-fit according to Cohen (1988). The VCI and WMI scores could not be predicted significantly. Table 4 shows the regression coefficients of the model for FSIQ and PSI, and Table 5 shows the regression coefficients of the model for VCI and WMI.

### 3.4. The Influence of the Number of Comorbid Diagnoses

For an overview of the two groups built by number of diagnoses, see Table 2. The MANOVA was performed with the independent variable “number of diagnoses” (two or fewer diagnoses vs. three or more diagnoses) and the dependent variables FSIQ, VCI, WMI and PSI. For descriptive statistics and results, see Table 6. No significant results were seen for any of the dependent variables.

## 4. Discussion 

In the present study, a group of adolescent inpatients diagnosed with SUD was compared to a healthy control group. Comorbid diagnoses were taken into account. The group of patients with SUD showed significantly lower scores than the control group in the VCI, WMI, PSI and the FSIQ. This stands in line with previous research with patients with SUD.

### 4.1. Verbal Comprehension

The results of a significantly lower VCI score stand in line with several studies that have linked cannabis use and alcohol use in adolescents to deficits in the verbal domain. Nguyen-Louie et al. (2016) [49] showed that more days with alcohol use predicted worse verbal memory. Further, in a study by Sullivan et al. (2021) on adult patients with SUD who were tested with the WAIS-IV [60], the VCI from the clinical group was below the mean relative to the population norm. Poorer verbal memory has been shown in adolescent alcohol drinkers (for a review, see [36]). Castellanos-Ryan et al. (2017) [43] found a lower verbal IQ in adolescent frequent cannabis users, but Latvala et al. (2009) [62] found that the lower IQ in adolescents with SUD was mainly explained by the participants’ parents or their own educational status. Here, the clinical group differed significantly from the control group in the VCI, but when only the clinical group was investigated, the VCI could not significantly be explained by the factors sex, education, a comorbid diagnosis of depression or the number of comorbid diagnoses. In a study by Hanson et al. (2011) [47], verbal learning deficits of cannabis users improved after three weeks of abstinence. Similarly, Schuster et al. (2018) [66] found improved verbal memory after one month of cannabis abstinence in a group of frequent adolescent cannabis users. At the time point of testing, the clinical group had been abstinent for at least 6 weeks—the verbal comprehension capacities in our inpatient group might therefore already have recovered at the time point of testing.

### 4.2. Working Memory

Here, the clinical group showed working memory impairments in the WMI compared to the control group. Working memory deficits have been shown for adolescent substance users in a wide range of studies (for a review, see [36]). Working memory impairments have also been shown for adolescent cannabis users (e.g., [44,67,68,69]). In a recent meta-analysis, Lorenzetti et al. (2019) [70] reported that regular adolescent cannabis users had smaller volumes in brain regions involved in learning and memory (e.g., hippocampus) compared to non-users. A similar picture appears for alcohol use in adolescents. Although one study showed that a lower working memory capacity before initiation of alcohol use could predict alcohol use in adolescents [71], Mahedy et al. (2018) [50] showed that a group of frequent alcohol consumers had a lower working memory performance compared to a non-alcohol-consuming group. In contrast to these findings, Sullivan et al. (2022) [60] found that adults with SUD did not show lower WMI scores compared to the population norm.

A model with the factors sex, type of education, comorbid diagnosis of depression and the number of comorbid diagnoses failed to predict the WMI outcome in our clinical sample.

### 4.3. Processing Speed Index

In this study, the clinical group showed worse performance on the PSI. This result conflicts with a study by Nguyen-Louie et al. (2015) [72]. While that study indicated that greater alcohol use by adolescents predicted worse psychomotor speed, processing speed was not predicted by substance use. Yet, similar to our findings, in a study by Sullivan et al. (2022), an adult clinical sample with SUD was found to have a lower PSI compared to the population norm [60]. Regarding cannabis, Fried et al. (2005) [69] found that current regular cannabis users performed worse in the domain of processing speed. In our clinical group, sex and a comorbid diagnosis of depression could predict the PSI. The negative influence of depression on processing speed is well known (for a meta-analysis, see [73]). The significant effect of the factor sex also stands in line with research showing that sex differences exist in processing speed, where women are faster in processing speed tasks involving digits and alphabets (as in the WISC-V/WAIS-IV subtest coding) (for a review, see [74]).

### 4.4. Full Scale IQ

Here, the lower FSIQ found for the clinical sample falls in line with findings from a large sample showing that cannabis use disorder is associated with lower fluid intelligence [45]. However, systematic reviews and meta-analyses show inconsistent results regarding the IQ of adolescent cannabis users (e.g., [39,75]). Jackson et al. (2016) [76] conducted two longitudinal twin studies and observed that cannabis use preceded a significant decline in crystallized intelligence between preadolescence and late adolescence. However, this decline might rather be attributable to familial factors underlying both cannabis use and a lower baseline IQ. Yet, a recent systematic review and meta-analysis found that cannabis exposure in youth led to a decline in IQ [77]. Since our data are cross-sectional, it remains an open question as to whether the lower IQ in our SUD sample preceded the substance use or whether it is one of the SUD’s negative consequences. The link between alcohol use and cognitive impairments seems to correlate with factors such as the frequency and the dose of alcohol consumption; for example, Hanson et al. (2011) [47] related worse cognitive functioning in adolescence to a heavier use pattern, worse hangovers and more extreme withdrawal symptoms (for a review, see [48]).

The level of education predicted the FSIQ in the clinical sample. This result stands in line with studies showing that intelligence scores and duration of education are positively correlated. Ritchie et al. (2018) [78] found meta-analytic evidence of positive effects of education on cognitive abilities.

### 4.5. Influence of the Number of Comorbid Diagnoses on Cognitive Abilities

No significant influence of the number of comorbid diagnoses on patients’ IQ scores was observed when we compared the group with one or two diagnoses to the group with three or more diagnoses. The group with one or two psychiatric diagnoses displayed higher IQ scores than the group with three or more psychiatric diagnoses, but this difference was not significant, possibly due to the small sample size. In a model to predict the outcome of the VCI, WMI, PSI and FSIQ, the number of comorbid diagnoses did not contribute to any of the variance. However, studies have linked a lower IQ in childhood to an increased risk for several common psychiatric disorders in adulthood as well as greater comorbidity (e.g., [79]).

### 4.6. Implications for Clinical Practice

In the current study, we observed a connection between adolescent substance abuse and lower cognitive capacity. However, we cannot make any unambiguous statements about the direction of this connection or causality. Adolescents with a lower IQ at baseline before their first substance use might be more prone to developing a SUD. Because studies have shown that after a SUD ceases, patients can improve several cognitive functions, it is recommended that inpatients with SUD should undergo retesting of their cognitive function after they have stopped taking drugs for a couple of months. Several studies show that cognitive deficits are common in SUD and predict a worse treatment outcome [80]. It might therefore be advisable to include intelligence testing as part of the standard diagnostic in SUD treatment and to include further neuropsychological tests to explore executive functions further, e.g., measures of attention or memory tests. There are authors that have proposed that interventions that improve cognitive functioning might also contribute to a more long-term successful treatment of the SUD [81]; for a review, see [82]. It might therefore be useful to include working memory training or training of executive functioning in the treatment of patients with SUD. Patients’ lower cognitive functions, e.g., in their working memory, have also to be considered in therapy, such that therapists might need to offer more repetitions due to working memory deficits.

### 4.7. Limitations and Directions for Future Research

We compared a group of adolescents with diagnosed SUD with a control group, whereby the clinical group’s cognitive abilities were tested during hospitalization for the SUD. The sample size in this study is rather small and the results might therefore be distorted. A follow-up study with a larger sample size is needed. As participants were only included in the sample when intelligence diagnostics with the WAIS-IV [52] or WISC-V [53] took place and testing of intelligence was only administered when clinically indicated, the sample here might be prone to a selection bias.

The temporal sequence of the SUD and the reasons for the observed lower IQ scores in the VCI, WMI, PSI and FSIQ remain unclear, as adolescents in the clinical group might have displayed lower scores before their first substance use. It is also unclear whether the observed effects remain stable over time or will change over longer periods; these questions and the mechanisms involved can only be answered in longitudinal designs, such as through a follow-up study with post-tests after a span of months and years. Another issue was that the examined clinical group displayed many comorbid diagnoses, with depression being the most common. To rule out possible distortions, future studies should include a control group of matched participants showing the same comorbid diagnoses but no SUD. Furthermore, research shows that an early onset of substance use (e.g., [43,51]) often leads to larger cognitive deficits. A further study might have to take this into account and determine how long participants’ substance use has occurred. A further confounding factor might be attention and impulsivity: several studies have linked substance use to a higher impulsivity and worse attention (see [36] for a review), which might also lead to worse results in a standardized test due to faster responses or more careless mistakes. Further studies should also test and control for impulsivity, attention and executive functions in patients with SUD.

## Figures and Tables

**Table 1:** Demographic description of the sample by group.

	WISC-V Clinical Group (*n* = 22)	WISC-V Control Group (*n* = 22)	WAIS-IV Clinical Group (*n* = 27)	WAIS-IV Control Group (*n* = 27)
Sex (*n* and % female)	10 (45.5%)	10 (45.5%)	12 (44.4%)	12 (44.4%)
M age (SD)	14.55 (0.80)	14.55 (0.80)	17.07 (0.98)	17.00 (0.06)
Type of education	*n* = 18	*n* = 22	*n* = 18	*n* = 27
Low	6 (33.3%)	2 (9.1%)	3 (16.7%)	4 (14.8%)
Middle	11 (61.1%)	19 (86.4%)	14 (77.8%)	22 (81.5%)
High	1 (5.6%)	1 (4.5%)	1 (5.6%)	1 (3.7%)

WISC-V: Wechsler Intelligence Scale for Children -Fifth Edition; WAIS-IV: Wechsler Adult Intelligence Scale-Fourth Edition; Low: secondary school, graduation after 9th grade (German: Hauptschule) or special school; Middle: middle secondary school, graduation after 10th grade (German: Realschule), comprehensive school forms (German: Gesamtschule), or professional schools (German: Fach-/Berufsschule); High: grammar school, graduation after 12th or 13th grade, university entrance degree (German: Gymnasium). Please note that for 13 participants, no demographic data were available.

**Table 2:** Number of patients with several comorbid diagnoses in the sample.

Number of Comorbid Diagnoses	0	1	2	3	4	5	6	7
N (total N = 49); %	0; 0%	8; 16.3%	13; 26.5%	10; 20.4%	12; 24.5%	4; 8.2%	1; 2.0%	1; 2.0%

**Table 3:** Mean and standard deviation and MANOVA results for FSIQ, VCI, WMI and PSI by group.

Index	Clinical Group *n* = 49		Control Group *n* = 49		MANOVA			
	M	SD	M	SD	*F*	(df1/df)	*p*	*η²*
FSIQ	85.71	8.89	96.77	12.35	25.882	(1/96)	<0.001	0.21
VCI	86.97	9.58	95.55	12.43	14.606	(1/96)	<0.001	0.13
WMI	90.14	12.38	97.47	15.10	6.897	(1/96)	0.010	0.07
PSI	91.59	12.71	97.86	13.57	5.559	(1/96)	0.020	0.06

FSIQ: Full Scale IQ; VCI: Verbal Comprehension Index; WMI: Working Memory Index; PSI: Processing Speed Index.

**Table 4:** Regression coefficients for predicting the FSIQ and the PSI.

Variable	FSIQ N = 36						PSI N = 36					
	B	SE [B]	95% CI	*β*	*t*	*p*	B	SE [B]	95% CI	*β*	*t*	*p*
Sex	1.27	2.86	[−4.57, 7.11]	0.072	0.444	0.660	11.05	3.85	[3.19, 18.91]	0.439	2.869	0.007
Type of education	8.28	2.71	[2.76, 13.80]	0.492	3.062	0.005	6.55	3.64	[−0.87, 13,98]	0.274	1.801	0.081
Depression	0.42	2.81	[−5.32, 6.12]	0.024	0.149	0.883	−8.89	3.79	[−16.63, −1.17]	−0.354	−2.347	0.025
Number of comorbid diagnosis	−0.79	0.99	[−2.83, 1.25]	−0.123	−0.788	0.437	−0.49	1.35	[−3.24, 2.25]	−0.054	−0.368	0.716
R^2^		0.31						0.38				
ΔR^2^		0.22						0.30				

CI = confidence interval for B. FSIQ: Full Scale IQ; PSI: Processing Speed Index.

**Table 5:** Regression coefficients for predicting the VCI and WMI.

Variable	VCI N = 36						WMI N = 36					
	B	SE [B]	95% CI	*β*	*t*	*p*	B	SE [B]	95% CI	*β*	*t*	*p*
Sex	−6.83	3.23	[−13.42, −0.23]	−0.365	−2.111	0.043	−0.58	4.45	[−9.66, 8.49]	−0.023	−0.131	0.896
Type of education	5.38	3.06	[−0.86, 11.61]	0.302	1.760	0.088	9.29	4.21	[0.72, 17.87]	0.383	2.210	0.035
Depression	5.55	3.18	[−0.93, 12.04]	0.297	1.746	0.091	−7.76	4.38	[−16.69, 1.17]	−0.304	−1.773	0.086
Number of comorbid diagnosis	−0.58	1.13	[−2.89, 1.71]	−0.087	−0.521	0.606	0.60	1.55	[−2.56, 3.78]	0.065	0.388	0.701
R^2^		0.21						0.19				
ΔR^2^		0.11						0.09				

CI = confidence interval for B. VCI: Verbal Comprehension Index; WMI: Working Memory Index.

**Table 6:** Differences in the index means between patients with one or two comorbid diagnoses or three or more diagnoses and results of MANOVA.

Index	Two or Fewer Comorbid Diagnosis N = 21		Three or More Comorbid Diagnosis N = 28		MANOVA			
	M	SD	M	SD	*F*	(df1/df)	*p*	*η²*
FSIQ	87.57	10.79	84.32	7.04	1.624	(1/47)	0.209	0.03
VCI	88.19	9.12	86.07	9.12	0.582	(1/47)	0.449	0.01
WMI	91.43	14.25	89.18	10.94	0.391	(1/47)	0.535	0.01
PSI	94.29	13.76	89.57	11.72	1.673	(1/47)	0.202	0.03

FSIQ: Full Scale IQ; VCI: Verbal Comprehension Index; WMI: Working Memory Index; PSI: Processing Speed Index. Here, significant group differences were seen for all indices, with the largest effects for the FSIQ and the VCI.

## Data Availability

Since this research is based on health service research (Versorgungsforschung), the data are not publicly available.

## Appendix A

**Table A1:** MANOVA results, means and standard deviations for the primary indices for the WISC-V and WAIS-IV by group.

**WISC-V Index**								
	Clinical Group		Control Group					
	M *n* = 22	SD	M *n* = 22	SD	*F*	(df1/df2)	*p*	*η²*
VCI	88.72	8.35	100.41	10.26	17.152	(1/42)	<0.001	0.29
VSI	87.91	11.55	100.59	11.54	11.482	(1/42)	0.002	0.21
FRI	92.18	13.28	99.45	13.83	3.163	(1/42)	0.083	0.07
WMI	94.81	13.18	99.81	14.90	1.388	(1/42)	0.245	0.03
PSI	93.86	14.01	102.41	15.82	3.594	(1/42)	0.065	0.07
FSIQ	87.31	9.73	100.00	14.05	12.100	(1/42)	0.001	0.22
**WAIS-IV Index**								
	*n* = 27		*n* = 27					
VCI	85.55	10.41	91.59	12.81	3.608	(1/52)	0.063	0.06
PRI	88.18	12.37	96.70	15.38	5.026	(1/52)	0.029	0.08
WMI	86.33	10.43	95.56	15.27	6.714	(1/52)	0.012	0.11
PSI	89.74	11.48	94.15	10.30	2.203	(1/52)	0.144	0.04
FSIQ	84.41	8.08	94.15	10.30	14.94	(1/52)	<0.001	0.22

FSIQ: Full Scale IQ; VCI: Verbal Comprehension Index; VSI: Visual Spatial Index; FRI: Fluid Reasoning Index; WMI: Working Memory Index; PSI: Processing Speed Index; PRI: Perceptual Reasoning Index. WISC-V: Wechsler Intelligence Scale for Children—Fifth Edition; WAIS-IV: Wechsler Adult Intelligence Scale—Fourth Edition.

In both the WISC-V and the WAIS-IV, the clinical group showed overall lower index scores than the control group. The control group achieved performances around the mean of the intelligence distribution (100 +/−15). Significant differences in FSIQ are found for both the group comparisons to WISC-V and WAIS-IV. In the analyses for the WISC-V, significant group differences were also found for the VCI and VSI. In addition, in the WAIS-IV, the performance on the PRI and WMI also differed significantly.
